# Current tobacco use and its associated factors among adults in a country with comprehensive ban on tobacco: findings from the nationally representative STEPS survey, Bhutan, 2014

**DOI:** 10.1186/s12963-016-0098-9

**Published:** 2016-08-08

**Authors:** Mongal Singh Gurung, Dorji Pelzom, Tandin Dorji, Wangchuk Drukpa, Chador Wangdi, Palanivel Chinnakali, Sonu Goel

**Affiliations:** 1 Ministry of Health, Thimphu, Bhutan; 2 Jawaharlal Institute of Postgraduate Medical Education and Research, Puducherry, India; 3 Post Graduate Institute of Medical Education and Research, Chandigarh, India

**Keywords:** Tobacco, Smoking, Prevalence, Smokeless tobacco, Bhutan, STEPS

## Abstract

**Background:**

Despite a comprehensive ban on cultivation, manufacture, distribution, and sale of tobacco products since 2004, two nationwide surveys conducted in 2012 and 2013 reported high tobacco use in Bhutan. National Health Survey 2012 reported that 4 % of the population aged 15–75 years used smoked tobacco and about 48 % used smokeless tobacco. Similarly, Global Youth Tobacco Survey (GYTS) of Bhutan reported tobacco use prevalence of 30.3 % in 2013. However, factors associated with this high tobacco use were not systematically studied. Hence, we assessed the prevalence of tobacco use and its associated sociodemographic, behavioral, and environmental factors.

**Methods:**

This cross-sectional analytical study used secondary data collected in a nationally representative Non-communicable Disease Risk Factors Surveillance STEPS Survey 2014 conducted among Bhutanese adults (18–69 years). The survey included a total of 2820 adults; selected using multistage stratified cluster sampling. Weighted analysis was done to calculate the prevalence of tobacco use. Unadjusted and adjusted prevalence ratios were calculated using log binomial regression.

**Results:**

The prevalence of current overall tobacco use was 24.8 % (95 % CI: 21.4–28.3) and that of smoked, smokeless, and dual forms (smoked and smokeless forms) were 7.4 % (95 % CI: 5.8–9.0), 19.7 % (95 % CI: 16.5–22.9), and 2.3 % (95 % CI: 1.8–2.9), respectively. Significantly higher prevalence of tobacco use in all forms was found among males, younger age groups, and alcohol users. The prevalence of smoked form was higher in urban areas compared to rural areas (11 % vs 6 %; aPR 1.8, 95 % CI: 1.5–2.0). Among individuals who reported having a non-communicable disease, the prevalence of smoked tobacco use was significantly lower than those who did not have disease (3.5 % vs. 8.3 %; aPR 0.5, 95 % CI: 0.3–0.9). Exposure to health warnings was protective for current tobacco use and smokeless tobacco use, while exposure to tobacco warnings through the media was helpful among smokers and overall tobacco users.

**Conclusions:**

Despite a comprehensive ban on tobacco, tobacco use was high in Bhutan, especially the smokeless form. Males, younger age groups, and alcohol users should be targeted with behavioral interventions along the stricter implementation of tobacco control measures.

## Background

Globally, tobacco use killed over 100 million people in 20^th^ century, and is projected to a take toll of one billion lives by the end of 21^st^ century [1]. According to a systematic analysis for the Global Burden of Disease Study 2013, the number of deaths attributable to tobacco smoking increased from 4.6 million in 1990 to 5.8 million in 2013 [2]. Similarly, the disability-adjusted life-years (DALYs) attributable to tobacco smoking increased from 115.9 million to 134.2 million DALYs over the same period. The same study reported that secondhand smoke accounted for an additional 331,000 deaths and 9.3 million DALYs. Another study reported that though there has been reduction in global prevalence of daily smoking from 1980 to 2012, the number of smokers has increased steadily [3]. In light of these findings, the World Health Organization (WHO) identifies tobacco as one of the important risk factors for non-communicable diseases (NCDs) and a major cause of premature death [1, 4, 5]. Further, use of tobacco is causing about half a trillion dollars of economic damage every year [1, 4]. In response, WHO developed the Framework Convention on Tobacco Control (FCTC), setting the foundation for reducing both demand for and supply of tobacco products. Based on WHO FCTC, countries have implemented partial or total restriction on availability and use of tobacco products [4].

Bhutan is the only country in the world which has a comprehensive ban on the cultivation, manufacture, distribution, and sale of tobacco products within the country under the Tobacco Control Act of 2010, further amended in years 2012 and 2014 [6]. It was also among the first few countries to have signed and ratified WHO FCTC in 2004 [7]. Because the cultivation and manufacture of tobacco was successfully banned in Bhutan, all tobacco used in the country is therefore imported.

Despite the ban and enactment of the Tobacco Control Act, the National Health Survey 2012 reported that 4 % of the population aged 15 to 75 years used a smoked form of tobacco, and about 48 % used smokeless tobacco [8]. Similarly, the Global Youth Tobacco Survey (GYTS) of Bhutan 2013 reported an increase in the prevalence of tobacco use, from 18.8 % in 2006 to 30.3 % in 2013 [9]. These two recent nationwide surveys clearly indicated the substantial burden of tobacco use in Bhutan. Hence the periodic assessment of prevalence of tobacco use and its associated factors will help in evaluating the tobacco control strategies and also guide in identifying the risk groups for prevention measures.

A recent study which used secondary data of the Global Adult Tobacco Survey (GATS) from four South Asian countries has identified the sociodemographic determinants of tobacco use [10]. Similarly, many nationwide studies across the globe have reported the association of demographic, behavioral, and environmental, or policy-related factors with tobacco use [10–24]. However, no literature exists in Bhutan which reports the determinants of tobacco use among adults. Hence, we aimed to determine the prevalence of tobacco use and its associated sociodemographic, behavioral, and environmental factors in a setting with a comprehensive ban on tobacco.

## Methods

### Study setting

Bhutan is a small landlocked country in South Asia with a projected population of 757,042 [25]. Administratively, Bhutan is divided into 20 Dzongkhags (districts), which are further divided into either Gewogs (blocks) for rural settings and Thomde (towns) for urban settings. The General Literacy Rate (2012) and Youth Literacy Rate (2012) are 63.0 % and 86.1 % respectively [25].

Bhutan has implemented a comprehensive ban on sale of tobacco products (both smoked and smokeless forms) since 2004 and put in place comprehensive smoke-free provisions since 2005. These tobacco control initiatives were further strengthened through the enactment of the Tobacco Control Act 2010 which provides for a comprehensive legal framework for the implementation of tobacco control policies and prohibits cultivation, manufacture, distribution, and sale of tobacco products (both smoked and smokeless forms) within Bhutan [26]. The law also governs smoke-free public places; tobacco advertising, promotion, and sponsorship; and requires that imported products bear the health warnings required in the country of origin. However, the law does not ban consumption of tobacco products, though it is banned in public places. A limited quantity of tobacco products may be imported by individual after paying tax for personal consumption [26].

The import of tobacco products for personal consumption with payment of sale tax and customs duty is allowed in Bhutan. Imports from India are levied a 100 % sales tax, and those from countries other than India are levied a 100 % sales tax and 100 % customs duty. The maximum quantity allowed per month per person is 800 sticks of cigarettes, 1200 sticks of bidis, 150 pieces of cigar, or 750 grams of tobacco and tobacco-related products. Documents required at the time of declaration are ID card, passport, voter card, or any other relevant document issued by his/her country and proof of purchase. The receipt issued is valid for one month. The receipt must be produced when required as proof of tax and duty paid. Any person 18 years and younger is not allowed to import tobacco products. A person shall not import tobacco products on behalf of another person [27].

### Study design and study population

A cross-sectional analytical study using the secondary data collected in a nationally representative Non-communicable Disease Risk Factors Surveillance STEPS Survey 2014 of Bhutan. Adults (18–69 years) constituted the study population.

### Sample size and sampling technique

The STEPS survey was a household sample survey which used multistage stratified cluster sampling technique to study risk factors for NCDs among adults. The sampling involved selection of primary sampling units (Gewogs in rural and towns in urban), secondary sampling units (Chiwogs in rural areas and enumeration areas in urban) followed by selection of households and individuals. A total of 2820 adults participated in the survey against 2912 adults approached (with a response rate of 96.8 %).

### Data variables and data sources

Independent variables included demographic characteristics (gender, age, marital status, place of residence, occupation, education), behavioral characteristics (alcohol, physical activity), environmental or policy-related characteristics (secondhand smoke at workplace or home, notice health warnings related to tobacco on media, advice by health workers on quitting, history of NCDs, family history of NCDs). The dependent variables were “current use of tobacco” and “current use of smoked and smokeless forms of tobacco.” Data on the above variables were extracted from the STEPS Survey dataset.

Current smokers were defined as persons who reported smoking any tobacco products, such as cigarettes, cigars, or pipes daily or non-daily irrespective of the quantity. Similarly, current smokeless tobacco users were defined as persons who reported using any smokeless tobacco such as snuff, chewing tobacco, or betel quid with tobacco daily or non-daily, irrespective of the quantity. Current tobacco use was defined as use of either smoked or smokeless form or both of tobacco.

### Data analysis

Data from STEPS survey were imported to STATA/IC 11 (Serial Number: 30110592257, StataCorp. 2009. *Stata Statistical Software: Release 11*. College Station, TX: StataCorp LP). Since the STEPS survey involved multistage sampling, we carried out a weighted analysis for calculating the prevalence of tobacco use and also in estimating the unadjusted and adjusted prevalence ratios. Population weight (post-sampling weight) was calculated to adjust for the difference in the age and gender distribution between the sample and population. For analysis, we used individual weights derived using the sampling weight and the population weight.

The categorical variables were summarized as proportions. Unadjusted prevalence ratios with 95 % confidence intervals were calculated to assess possible association of factors with smoked, smokeless, and overall tobacco use. Those factors which were significant in bivariate model at *P*-value < 0.1 were included in log binomial regression model to calculate adjusted prevalence ratio (aPR) with 95 % confidence intervals. *P* value of less than 0.05 was considered as statistically significant.

## Results

A total of 2820 adults were included in the study, of which 1748 (62 %) were females, 1766 (63 %) had no formal education, and 1952 (69 %) were from rural areas. Sociodemographic, behavioral, and environmental characteristics of participants are shown in Table 1. Of 2820 participants, 2201 (78 %) had reported exposure to health warnings related to tobacco use through media, 1148 (41 %) had reported receiving health advice related to tobacco use from a health care provider, and 609 (22 %) reported having an NCD. Alcohol use in last 12 months was reported by 1360 (48 %) participants.

**Table 1:** Factors associated with current tobacco use among adult Bhutanese aged 18-69 years, STEPS Survey, 2014

Characteristics	Total	Tobacco use (%)^a^	PR (95 % CI)^a^	Adjusted PR (95 % CI)^a^
Total	2820	24.8	-	-
Age groups				
18–24	281	23.4	1.2 (1.1–1.2)	1.6 (1.2–2.1)
25–34	762	29.1	1.5 (1.4–1.5)	1.5 (1.2–1.8)
35–44	751	24.9	1.3 (1.2–1.3)	1.1* (0.9–1.4)
45–54	572	19.9	Ref	Ref
55–69	456	20.2	1.0 (1.0–1.0)	1.0* (0.7–1.3)
Gender				
Male	1074	33.6	2.5 (2.4–2.5)	2.4 (2.0–3.0)
Female	1748	13.6	Ref	Ref
Highest level of education				
Secondary	421	26.7	1.3 (1.2–1.3)	1.2* (1.0–1.4)
Primary	632	31.8	1.5 (1.5–1.5)	1.3 (1.1–1.5)
No formal schooling	1766	21.0	Ref	Ref
Occupation				
Employed	480	29.8	1.9 (1.8–1.9)	0.9* (0.7–1.2)
Self employed	1558	26.8	1.7 (1.7–1.7)	1.3 (1.1–1.6)
Unpaid/student/homemaker	782	15.8	Ref	Ref
Marital status				
Never married	225	22.9	0.8 (0.8–0.9)	0.5 (0.4–0.7)
Married/co-habiting	2278	24.8	0.9 (0.9–0.9)	0.7 (0.6–0.9)
Separated/widowed/divorced	317	27.9	Ref	Ref
Alcohol use				
Yes	1,360	32.6	1.9 (1.9–1.9)	1.8 (1.5–2.0)
No	1,459	17.1	Ref	Ref
Secondhand smoke exposure				
Yes	777	32.3	1.5 (1.5–1.5)	1.2 (1.1–1.3)
No	2045	21.5	Ref	Ref
Exposure to tobacco warnings				
Yes	2201	24.1	0.9 (0.9–0.9)	0.9 (0.7–1.0)
No	619	27.81	Ref	Ref
Family history of NCDs^b^				
Yes	1247	27.1	1.2 (1.2–1.2)	1.2 (1.1–1.3)
No	1572	23.0	Ref	Ref
Physical activity^c^				
Yes	2417	26.1	Ref	Ref
No	402	16.4	0.6 (0.6–0.7)	0.7 (0.6–0.9)

Only significant variables in the multivariate model are shown in table, other variables included in the analysis were history of NCDs, place of residence (rural vs. urban), and advice by health workers

^a^Weighted analysis, PR- unadjusted prevalence ratio, NCD- Non-communicable diseases

^b^Blood family members (sibling, parent, grandparent, aunt or uncle) been diagnosed with following; diabetes or raised blood sugar, raised blood pressure, stroke, cancer or malignant tumor, raised cholesterol, early heart attack, asthma or chronic lung disease (COPD) or kidney disease

^c^Work involves vigorous/moderate-intensity activity that causes large increases in breathing or heart rate

*Not significant at *P*-value <0.05

The prevalence (weighted) of current tobacco use (smoked or smokeless form) was 24.8 % (95 % CI: 21.4–28.3). Factors associated with current tobacco use are shown in Table 1. Significantly higher prevalence was found among males, younger age groups, literate, alcohol users, individuals who were exposed to secondhand smoke, and among those who were widowed or separated. There was no statistically significant difference in prevalence of tobacco use between urban and rural areas (23.7 % vs. 25.3 %). Prevalence of tobacco use was higher among individuals who were not exposed to tobacco warnings in media compared to those who were exposed (28 % vs. 24 %) and this difference was statistically significant (*P* < 0.001).

The prevalence (weighted) of current use of smoked form of tobacco was 7.4 % (95 % CI: 5.8–9.0). Factors associated with current use of smoked form of tobacco are shown in Table 2. Males, younger age groups, the literate, alcohol users, and individuals who were exposed to secondhand smoke had higher prevalence. The prevalence was higher in urban areas compared to rural areas (11 % vs. 6 %), and the difference was statistically significant (aPR 1.7, 95 % CI: 1.3–2.1). Among individuals who reported having a NCD, the prevalence was lower compared to those who did not (4 % vs. 8 %) and the difference was statistically significant (aPR 0.5, 95 % CI: 0.3–0.9).

**Table 2:** Factors associated with current smoked form of tobacco use among adult Bhutanese aged 18–69 years, STEPS Survey, 2014

Characteristics	Total	Current Smokers (%)^a^	PR (95 % CI)^a^	Adjusted PR (95 % CI)^a^
Total	2822	7.4	-	-
Age groups (years)				
18–24	281	11.3	3.6 (3.4–3.9)	3.0 (1.5–6.2)
25–34	762	11.7	3.7 (3.5–4.0)	2.9 (1.5–5.5)
35–44	751	4.1	1.3 (1.2–1.4)	1.0* (0.5–2.2)
45–54	572	3.1	ref	Ref
55–69	456	3.7	1.2 (1.0–1.3)	0.5* (0.1–2.1)
Gender				
Male	1074	10.8	3.5 (3.3–3.6)	3.3 (2.2–5.0)
Female	1748	3.1	Ref	Ref
Highest level of education				
Secondary	421	15.2	3.4 (3.3–3.5)	1.7 (1.3–2.3)
Primary	632	8.4	1.9 (1.8–1.9)	1.0 (0.7–1.3)
No formal schooling	1766	4.5	Ref	Ref
Place of residence				
Urban	870	11.1	1.9 (1.9–2.0)	1.7 (1.4–2.2)
Rural	1952	5.8	Ref	Ref
Marital status				
Never married	225	11.2	1.9 (1.7–2.0)	0.6 (0.3–1.0)
Married/co-habiting	2278	7.1	1.2 (1.1–1.3)	0.7* (0.5–1.2)
Separated/widowed/divorced	317	6.0	Ref	Ref
Occupation				
Employed	480	11.6	3.1 (2.9–3.2)	0.8* (0.6–1.2)
Self employed	1558	7.3	1.9 (1.9–2.0)	1.8 (1.2–2.6)
Unpaid/student/homemaker	782	3.8	Ref	Ref
Alcohol use				
Yes	1,360	9.4	1.5 (1.2–1.9)	1.5 (1.2–1.9)
No	1,459	5.5	Ref	Ref
Exposure to secondhand smoke				
Yes	777	13.5	1.7 (1.4–2.2)	1.7 (1.4–2.2)
No	2045	4.7	Ref	Ref
Advice by health workers				
Yes	1148	6.6	0.8 (0.6–0.9)	0.8 (0.7–0.9)
No	1671	8.0	Ref	Ref
History of NCDs^b^				
Yes	609	3.5	0.6 (0.4–1.0)	0.5 (0.3–0.9)
No	2210	8.3	Ref	Ref

Only significant variables in the multivariate model are shown in this table; other variables included in the analysis were family history of NCDs, and physical activity

^a^Weighted analysis, PR- unadjusted prevalence ratio, NCD- Non-communicable diseases

^b^Self-reported history of raised blood pressure, diabetes, raised total cholesterol, and cardiovascular diseases

*Not significant at *P*-value <0.05

Regarding current use of smokeless form of tobacco, the weighted prevalence was 19.7 % (95 % CI: 16.5–22.9). Prevalence in different subgroups is shown in Table 3. Prevalence was more than twice higher among males compared to females (aPR 2.5, 95 % CI: 2.0–3.1). Similarly, higher prevalence was found among alcohol users compared to non-users (aPR 1.9, 95 % CI: 1.6–2.3). As shown in Table 4, among the current smokeless tobacco users, chewing tobacco/snuff by mouth were the most widely used (95.5 %) types of smokeless tobacco, followed by betel quid with tobacco (6.1 %), and snuff by nose (3.7 %). Prevalence of current use of both forms of tobacco (smoke and smokeless forms) was 2.3 % (95 % CI: 1.8–2.9).

**Table 3:** Factors associated with current smokeless tobacco use among adult Bhutanese aged 18–69 years, STEPS Survey, 2014

Characteristics	Total	Current Smokeless Tobacco user (%)^a^	PR (95 % CI)^a^	Adjusted PR (95 % CI)^a^
Total	2820	19.7	-	-
Age groups				
18–24	281	16.6	0.9 (0.9–1.0)	1.8 (1.4–2.5)
25–34	762	20.8	1.2 (1.1–1.2)	1.5 (1.2–1.8)
35–44	751	22.2	1.3 (1.2–1.3)	1.2* (0.9–1.5)
45–54	572	17.8	Ref	Ref
55–69	456	17.2	1.0 (0.9–1.0)	0.9* (0.6–1.2)
Gender				
Male	1074	26.5	2.4 (2.4–2.5)	2.7 (2.1–3.4)
Female	1748	11.0	Ref	Ref
Highest level of education				
Secondary	421	15.2	0.8 (0.8–0.8)	0.9 (0.7–1.1)
Primary	632	25.5	1.4 (1.4–1.4)	1.3* (1.1–1.5)
No formal schooling	1766	18.5	Ref	Ref
Marital status				
Never married	225	16.5	0.8 (0.7–0.8)	0.5 (0.3–0.7)
Married/co-habiting	2278	19.9	0.9 (0.9–0.9)	0.8* (0.6–1.1)
Separated/widowed/divorced	317	22.2	Ref	Ref
Alcohol use				
Yes	1,360	27.1	2.2 (2.2–2.2)	2.2 (1.9–2.7)
No	1,459	12.3	Ref	Ref
Exposure to tobacco warnings in general				
Yes	2201	18.5	0.8 (0.7–0.8)	0.8 (0.7–0.9)
No	619	24.6	Ref	Ref
Family history of NCDs^b^				
Yes	1247	21.1	1.1 (1.1–1.2)	1.2 (1.1–1.4)
No	1572	18.6	Ref	Ref
Physical activity^c^				
Yes	2417	9.7	Ref	Ref
No	402	21.3	0.5 (0.4–0.5)	0.6 (0.4–0.8)

Only significant variables in the multivariate model are shown in table, other variables included in the analysis were history of NCDs, place of residence (rural vs. urban), occupation, and advice by health workers

^a^Weighted analysis, PR- unadjusted prevalence ratio, NCD- Non-communicable diseases

^b^Blood family members (sibling, parent, grandparent, aunt or uncle) been diagnosed with following; diabetes or raised blood sugar, raised blood pressure, stroke, cancer or malignant tumor, raised cholesterol, early heart attack (below age 50 for men and below age 55 for women), asthma or chronic lung disease (COPD) or kidney disease

^c^Work involves vigorous/moderate-intensity activity that causes large increases in breathing or heart rate

*Not significant at *P*-value <0.05

**Table 4:** Types of smokeless tobacco used by the current users of smokeless tobacco, STEPS Survey, 2014 (Both Sexes)

Age Group (years)	n	% Chewing tobacco/Snuff by mouth^a^	95 % CI	% Snuff by nose^a^	95 % CI	% Betel quid with tobacco^a^	95 % CI	% Other^a^	95 % CI
18–39	227	96.7	94.2–99.3	4.5	0.0–9.3	3.0	0.8–5.2	3.7	1.1–6.3
40–69	222	92.9	88.7–97.1	1.9	0.2–3.7	12.5	6.2–18.7	2.4	0.0–4.8
18–69	449	95.5	93.2–97.8	3.7	0.3–7.0	6.1	3.3–8.9	3.3	1.2–5.3

^a^Weighted analysis

## Discussion

This nationally representative survey found that one-fourth of adults in Bhutan use tobacco, with the majority of those using smokeless forms of tobacco. The prevalence was higher among men, younger age groups, and alcohol users. Among those who had received health advice regarding tobacco use or seen tobacco warnings through media, the prevalence was low.

One of the possible reasons for more Bhutanese using smokeless tobacco could be because of the smoking ban in public places. It is convenient for the people to use smokeless tobacco, as they don’t have to visit the “designated smoking room.” Moreover, other people usually don’t mind smokeless tobacco use, as it doesn’t produce secondhand exposure. Affordability could be another reason for more people, particularly from the poorer section of society, using smokeless tobacco in Bhutan.

The following are the strengths of the study. First, this is the first nationwide study exploring the factors associated with current tobacco use among adults in Bhutan. Second, we have comprehensively assessed the factors including environmental factors like exposure to health advice, tobacco warnings, history of NCDs in the individual and family. Third, since the non-response rate was very low (3 %) and we used weighted analysis to adjust for the complex survey design, the findings can be generalized to the whole of Bhutan. Finally, we adhered to Strengthening the Reporting of Observational Studies in Epidemiology (STROBE) guidelines in conducting and reporting the study [28].

In our study, the prevalence of current tobacco use was 25 %, while the prevalence of smoked tobacco was 7.4 %, and that of smokeless form was 19.7 %. Bhutan’s National Health Survey of 2012, which studied the age group from 10–75 years, reported usage of smoked and smokeless forms of tobacco as 4 % and 48 % respectively [8]. Though there seems to be an increase in use of smoked form, the difference in age groups studied makes it difficult to compare the two survey findings. Similarly, the definition of smokeless form in the current study excluded betel quid without tobacco, and this may be a reason for the marked difference in prevalence of smokeless form between the two surveys. Similar to Bhutan, the prevalence of smokeless form of tobacco was reported to be higher in India, Bangladesh, Indonesia, and Thailand as compared to smoked form of tobacco, while it is the other way round in many other countries [19].

A study based on GATS carried out between 2009–2011 in South Asian countries reported tobacco use prevalence of 43 % in Bangladesh, 36 % in Indonesia, and 35 % in India [10]. Compared to those countries, the prevalence of tobacco use in Bhutan is low. However, some other countries like Mexico (16 %), Egypt (20 %), and UK (23 %) have lower prevalence than Bhutan [19, 29]. In the current study, the prevalence of smoked form was higher in urban areas compared to rural areas and the finding is in contrast to those reported in other studies and tobacco-related surveys [10, 12]. Also, tobacco use was higher among males and younger age groups. These findings are similar to that of Global Youth Tobacco Survey and National Health Survey of Bhutan; however, our findings showing higher prevalence in the younger age group contradicts the findings from the South Asia [8, 10, 12, 30].

Studies have reported that education is an important determinant of tobacco use [10, 12, 13]. Our study found that use of smoked form of tobacco was common among educated compared to those who did not have formal education. However, with regard to use of smokeless form, the prevalence among those who only had primary education was higher than the prevalence seen in those with secondary education or more and those who did not have formal education. Our findings are similar to the findings from GATS, Thailand, whereas in other South Asian countries the prevalence was higher among the no formal education group [10]. As expected, the prevalence of tobacco use was about twice as high among alcohol users, as people who drink often smoke and vice versa. Similar associations have been reported in the previous studies [31].

Several studies have reported the impact of exposure to tobacco-related health warnings in media on tobacco initiation and quitting [32]. Our study findings also support this evidence. The prevalence of tobacco use was 20 % lower among those who had been exposed to health warnings. Similarly, health advice through health care workers also had an impact as the prevalence was found to be low among who were advised and this has been reported in previous studies [33].

The limitations of the study were that as the data was from a cross-sectional survey, we could not establish a temporal relationship between the associated factors and tobacco use. In addition, the possibility of social desirability bias in reporting tobacco use and alcohol use might have led to underestimating of prevalence. Since, the survey was carried out by health care workers, social desirability might be even higher.

The study has few implications. First, considering that overall tobacco use of 25 % is surprisingly high in a country where there is comprehensive ban on tobacco, control measures including ban on procurement, sale, and use in public places should be strictly implemented. Second, as tobacco use is a behavioral problem and health advice is proven to be useful, importance should be given to educate the patients of any non-communicable disease during their contact with health care provider. Further, the public or high-risk groups should be educated through conventional and folk media. Third, the higher tobacco use among younger age group is a concern, as it clearly indicates high tobacco initiation in younger age group. Targeted interventions in educational institutions (schools and colleges) should be initiated and strengthened. Fourth, tobacco cessation services can be strengthened at primary care level and its access improved for those who want to quit. Primary care health workers, nurses and teachers can be trained in counselling. Finally, alcohol use and tobacco use go hand in hand [34]. Similar to the ban on tobacco, restrictions on alcohol sale might help in reducing tobacco use.

## Conclusions

Tobacco use was high in Bhutan especially the smokeless form. Males, younger age group and alcohol users should be targeted with behavioral interventions for reducing the tobacco use along the stricter implementation of tobacco control measures.

## Abbreviations

aPR, adjusted prevalence ratio; CI, confidence interval; FCTC, Framework Convention for Tobacco Control; NCD, non-communicable diseases; REBH, Research Ethics Board of Health; WHO, World Health Organization
